# Development and validation of a nitrogen dioxide passive sampler

**DOI:** 10.1016/j.mex.2023.102334

**Published:** 2023-08-23

**Authors:** Elahe Hasannezhad Estiri, Abolfazl Rahmani Sani, Afshin Dowlatabadi, Reza Hasannezhad Estiri, Mohammad Miri

**Affiliations:** a Student Research Committee, Department of Environmental Health, School of Public Health, Sabzevar University of Medical Sciences, Sabzevar, Iran; b Non-Communicable Diseases Research Center, Department of Environmental Health Engineering, School of Public Health, Sabzevar University of Medical Sciences, Sabzevar, Iran.; c Environmental Science and Technology Research Center, Department of Environmental Health, School of Public Health, Shahid Sadoughi University of Medical Sciences, Yazd, Iran; d Department of Civil Engineering, Islamic Azad University Of Sabzevar, Sabzevar, Iran.; e Lishmaniose Research Center, Department of Environmental Health Engineering, School of Public Health, Sabzevar University of Medical Sciences, Sabzevar, Iran.

**Keywords:** Ambient air, NO_2_, Passive sampling, Trafic related air pollution, NO2 passive sampling

## Abstract

Nitrogen dioxide (NO_2_) is one of the main indicators of traffic-related air pollution in urban areas. Active sampling methods (common methods) are expensive and need advanced devices. While Passive sampling is a simple and low-cost method for measuring air pollutants, including NO_2_. Therefore in this study, we developed a passive sampler to measure ambient NO_2_ and validation its performance by comparing it with active sampling methods. Ambient NO_2_ was measured for 24 h by both active and passive sampling methods in the same locations (2 m height above grand level and 15 m distance from air pollution sources). Sampling of NO_2_ was repeated for 18 days to compare ambient NO_2_ concentrations measured by active and passive methods and validation our developed passive samplers.

• To develop passive samplers we used three stainless steel filters impregnated with a combination of triethanolamine and acetone (25:25 mL) in each tube.
• Active NO_2_ sampling was conducted using the modified Satlzman method (standard method).
• There was a strong correlation between NO_2_ concentration obtained from active and passive sampling methods (r = 0.84).

## Graphical abstract

**Figure:**
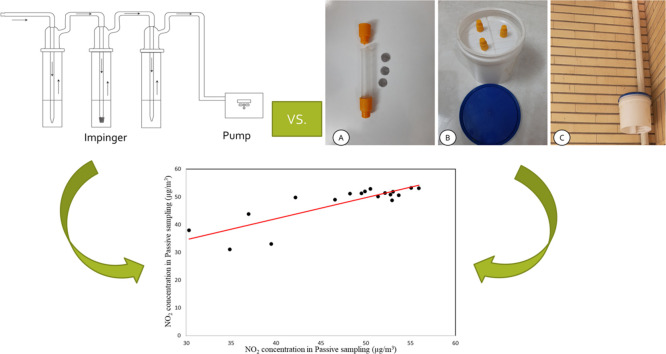

Specifications table

**Table:** 

Subject area:	Environmental Science
More specific subject area:	*Environmental Exposure Assessment, Environmental Health, Environmental Epidemiology*
Name of your method:	*NO2 passive sampling*
Name and reference of original method:	*Saltzman method*[1]
Resource availability:	*NA*

## Method details

This stud was conducted in Sabzevar, Iran (2022). We developed a simple passive sampler to measure ambient NO2 in urban areas. The performance of the developed passive sampler was evaluated by comparing it with a standard method for measuring NO2 [1].

## Preparation of passive sampling

**Fig. 1:**
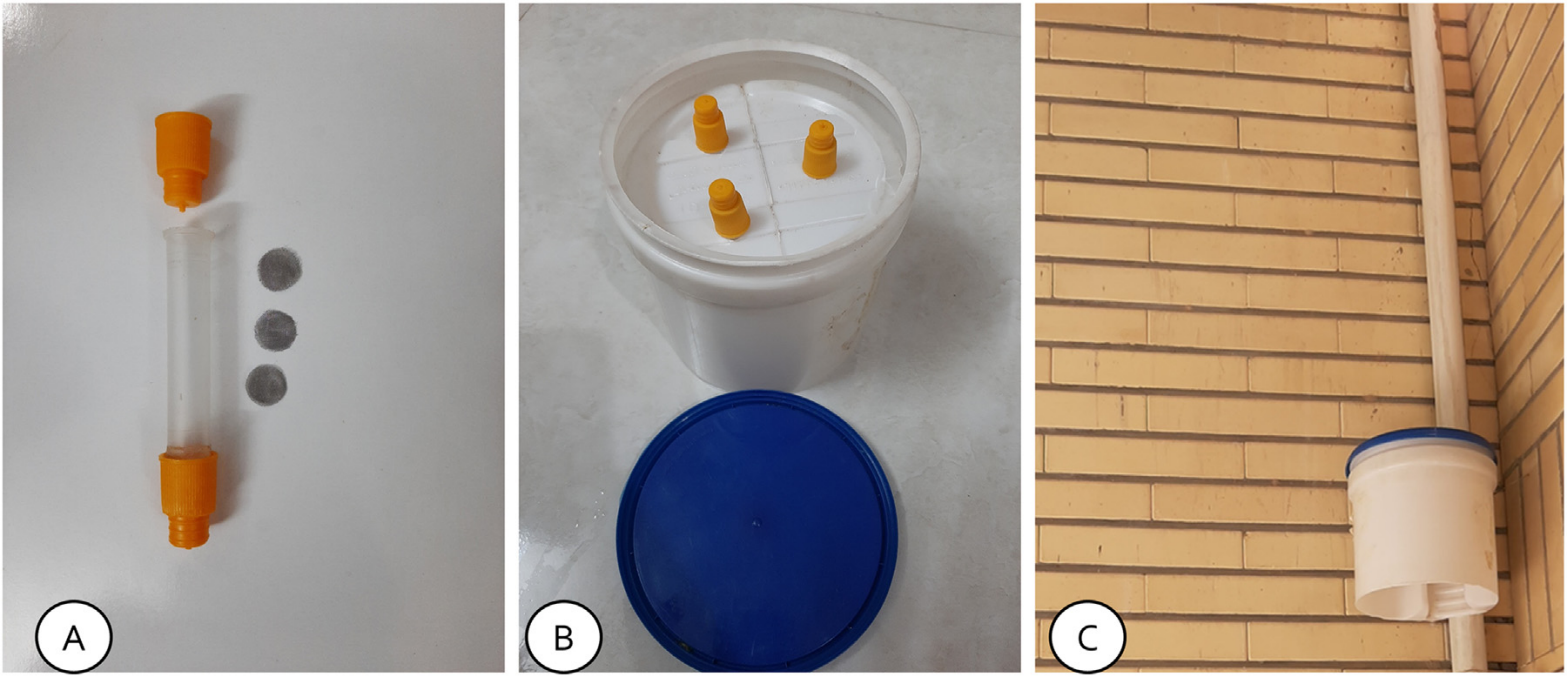
Developed passive sampler (A: a diffusion tube and 3 stillness still filters; B: prepared shelter and installed 3 tubes for smaller; C: installed sampler on 2 m height from ground level in the urban area).

In the preparation of the passive sampler, three stainless steel filters were immersed in an absorbent solution consisting of a mixture of 25 mL of triethanolamine and 25 mL of acetone for a duration of 20 min, as detailed in references [2], [3], [4], Subsequently, the filters were dried in a controlled environment and carefully positioned at the end of a polyethylene tube [5]. This polyethylene tube was specifically designed for passive sampling purposes. The end of the tube where the filters are situated was securely sealed with a specialized lid, while the opposite end of the tube was left open to facilitate air intake upon installation of the samplers in the urban area. To prevent direct sunlight and weather effects on the samplers, a shelter was designed and the samplers were placed inside it [6]. Notably, each sampler was equipped with three tubes, a configuration chosen to enhance the precision and reliability of the outcomes (Fig. 1).

In brief, the following chemicals and equipment were used to measure nitrogen dioxide in the air using the passive method [7]:

(1) Polyethylene diffusion tubes with a length of 7.1 cm and a diameter of 1 cm [8,9]
(2) stillness still filters with a mesh size of 150 µm and a diameter of 1 cm [10]
(3) Distilled water
(4) Sulfanilamide
(5) Orthophosphoric acid
(6) N-(1-naphthyl) ethylenediamine dihydrochloride (NEDA)
(7) Ethanolamine
(8) Acetone

All chemical compounds utilized in the study were of laboratory grade and were sourced from Merk Company, Germany.

### Laboratory analysis and NO_2_ calculation of samples obtained from the passive method

Initially, the filters were extracted from the polyethylene tubes and transferred to individual test tubes. Subsequently, a combination of reagents 1 and 2, mixed in a 1:1 proportion, was introduced, with 4 mL of the resultant solution added to each test tube containing the passive samplers. During this stage, the test tubes were positioned on a shaker for a duration of 30 min to guarantee thorough absorption [11]. Finally, the extent of absorption was quantified using a spectrophotometer (DR5000) operating at a wavelength of 510 nm.

#### Calculation of passive sampling

To determine the concentration of NO_2_ in the air under standard conditions (at a temperature of 293 K and a pressure of 101.3 kpa), the following formula was used [7]:

(1) C=(T/293)×m/(Srate×t)

C = Concentration of NO_2_ in the atmosphere (µg/m^3^)
T = Mean monthly temperature (° K)
m = Mass of NO_2_ in the passive sampler
Srate = Sampling rate (m^3^/h)
t = Sampling time (h)

To calculate the “m” and “Srate” the following formula was used: the equation extracted from the calibration curve of the spectrophotometer, which is expressed in formula-5, was used.

(2) x=(y−0.2394)/(−0.0453)

Y: Mean absorption (µg)
X: Nitrite present in the absorbent solution of the passive sampler
This equation is extracted from the calibration curve of the spectrophotometer (Fig. 3).

To calculate the Srate, first, the dispersion coefficient of NO_2_ gas should be calculated according to following formula [7]:

(3) Dt−month=D273×(t−month/273)1.81=0.136cm2s−1×(t−month/273)1.81

Dt-month: Gas dispersion coefficient based on the monthly mean temperature (cm^2^/s)
273D: Gas dispersion coefficient
T – month: monthly mean temperature (° k)

Regarding the gas dispersion coefficient, Massman [12] demonstrated in 1998 that the best estimate under standard conditions, is 0.136 cm^2^/s. Finally, the Srate was measured as follow [12]:

(4) Srate=(Dt−month×a)/L

a = Area of the passive sampling tube (m^2^)
L = Length of the passive sampling tube

## Sampling of ambient NO_2_ by the active method

In the assessment of ambient NO_2_ through the active approach, the adapted Saltzman method was employed, relying on the absorption process within a solution containing sodium hydroxide and sodium arsenite. In this procedure, the concentration of absorbed nitrite ions during sampling is gauged via colorimetry. This is achieved by subjecting nitrite ions to a reaction with phosphoric acid, sulfanilamide, and N-(1-naphthyl) ethylenediamine dihydrochloride (NEDA), followed by the measurement of absorbance of the resultant azo-dye at 510 nm [13], [14], [15].

The required chemical compounds for preparing the solutions in this stage and also analyzing the samples obtained from the active method are as follows:

• Distilled water
• Sodium hydroxide
• Sodium arsenite
• Sulfanilamide: with a melting point of 157 to 165°C
• N-(1-naphthyl) ethylenediamine dihydrochloride (NEDA)
• 30% hydrogen peroxide
• 85% phosphoric acid

To prepare the required solutions, the following method was used:

(1) Preparation of the absorbent solution: 4 g of sodium hydroxide was dissolved in distilled water, and 1 g of sodium arsenite was added to it. Then, it was brought to a volume of 1000 mL in a volumetric flask.
(2) Sulfanilamide solution: 20 g of sulfanilamide was dissolved in 700 mL of distilled water, and 50 mL of 85% phosphoric acid was added to it while stirring. Then, it was brought to a volume of 1000 mL in a volumetric flask. This solution is stable in the refrigerator for one month.
(3) NEDA solution: 0.5 g of NEDA was dissolved in 500 mL of distilled water. The stability of this solution extends for one month when stored in a refrigerator and shielded from light.
(4) Hydrogen peroxide solution: To obtain this solution, 2.0 mL of 30% hydrogen peroxide was diluted in 250 mL of distilled water. The stability of this solution extends for one month when stored in a refrigerator and shielded from light.

The calibration of the SKC SAMPLING PUMP 901 - 2011 was carried out using a soap bubble set, with meticulous consideration of the sampling conditions (with two standard nozzle impingers and one fritted nozzle impinger connected in series contained 10 mL of absorbent solution), encompassing the pressure drop induced by the placement of impingers along the pump's path. Calibration involved employing three impingers, mirroring the sampling conditions, and during this configuration, the air inflow into the pump was adjusted to 142 mL/min. A bypass valve was used to adjust the flow rate.

Upon connecting the impingers to the environmental pump, operating at a flow rate of 142 mL/min, a 24 h sampling was conducted at a height of 2 m from ground level. Following the completion of sampling, the absorbent solution was transferred from the impingers into a graduated vial and maintained at a temperature of 4  C until analysis, ensuring the preservation of the cold chain [13,14].

### Laboratory analysis of samples obtained from the active method

In the first step, the evaporation-induced reduction in the absorbent solution was rectified by introducing distilled water into the sampling process. Following thorough mixing, a 10 mL portion of the amassed sample was dispensed into a 50 mL volumetric flask. Subsequently, 1 mL of hydrogen peroxide solution, 10 mL of sulfanilamide solution, and 4.1 mL of NEDA solution were incorporated into the volumetric flask, with the volume being adjusted to 50 mL using distilled water. After a 10-min color development period, the magnitude and absorption characteristics of the sample were gauged using a spectrophotometer (DR5000) at a wavelength of 510 nm. To mitigate potential sources of error such as chemical and environmental contamination, a control sample was devised by employing the same sample preparation methodology. This involved transferring 10 mL of an absorbent solution that had not been exposed to the ambient air into a vial, followed by the continuation of the remaining sample preparation procedures as delineated earlier [13,14].

### NO_2_ calculations for the active method

To determine the concentration of NO_2_ obtained from the active method, the volume of sampled air was first converted to the volume under standard conditions (293 K and 101325 pa) using Formula 5. The average temperature and pressure were obtained from the National Meteorological Organization and applied to the equations [15].

(5) Vr=(VxP/101.3)x(298.15/T)

Where:

Vr = the volume of sampled air under standard conditions (L)
V = the volume of sampled air under ambient conditions (L)
P = the average atmospheric pressure at the sampling location (kPa)
T = the average atmospheric temperature at the sampling location (° K)
1.01325 = standard atmospheric pressure in kilopascals
298 k= standard atmospheric temperature in Kelvin

Next, we calculate the concentration of NO_2_ in the sample using the following formula [15]:

(6) C=(Axmx103xv)/Vr

C = concentration of nitrogen dioxide in the atmosphere (µg/m^3^)
A = absorbance (obtained from the spectrophotometer)
m = calibration factor (µg of NO_2_ per mL of absorbent solution per absorbance unit)
Vr = the volume of sampled air under standard conditions (L)
103 = conversion factor from liters to cubic meters
v = volume of the absorbent solution used in the reaction (mL)
It should be noted that the “m” was measured as described in formula 2.

## Calibration of the spectrophotometer (DR 5000) to measure NO_2_

To calibrate the spectrophotometer before analyzing the samples, a wide range of nitrite calibration standards were prepared. To prepare the standard solutions, 2 g of sodium nitrite was placed in an oven at 102  C for 2 h to prepare the stock solution. Then, the plate containing the dried sodium nitrite was placed in a desiccator for 15 min, and then 1.5 g of it was weighed and brought to one liter with distilled water in a one-liter flask. Standards of 15, 30, 60, 90, and 100 µg/mL were prepared by the following method [7]:

(1) 10 g of sulfanilamide was transferred to a 500 mL flask along with 25 mL of orthophosphoric acid, and then brought to volume.
(2) 70 mL of NEDA was transferred to a 500 mL flask and brought to volume.

**Fig. 2:**
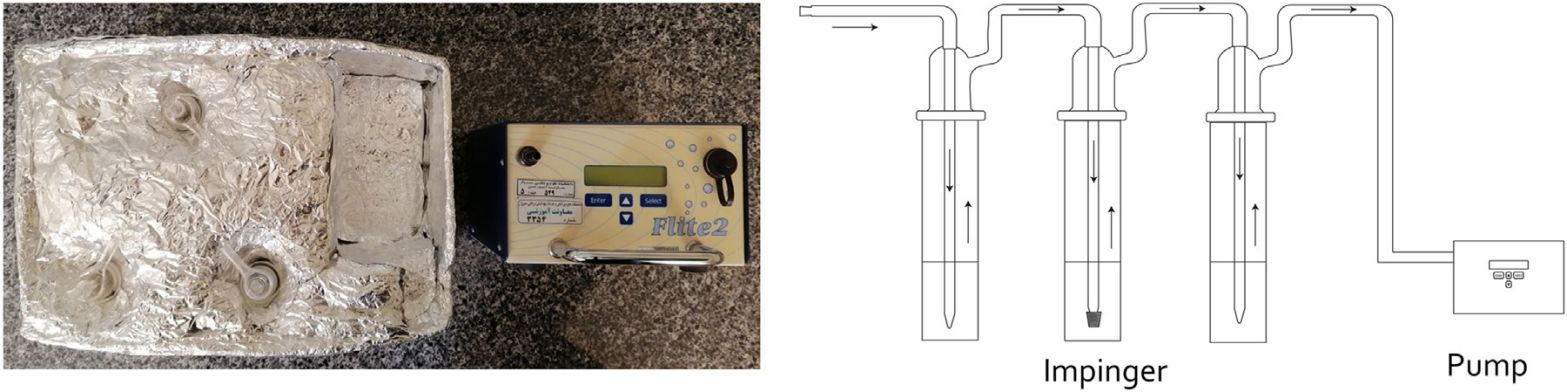
Series impingers to measure NO2 by the active method.

Subsequently, the generation of standards involved the amalgamation of equivalent quantities of the aforementioned solutions, and 4 mL of the resultant solution was incorporated into each of the formulated standards. The absorption characteristics of these standards were subsequently ascertained at a wavelength of 510 nm employing a spectrophotometer. As previously indicated, the concentration determination is predicated on the degree of nitrite ion absorption during the sampling process utilizing colorimetry, which hinges upon the interaction of nitrite ions with phosphoric acid, sulfanilamide, and N-(1-naphthyl) ethylenediamine dihydrochloride (NEDA). Hence, the more diminished the nitrite concentration within the solution, the less intricate the resultant compound formed with the reagent, yielding a solution with a lighter hue. Consequently, an increase in nitrite concentration within the sample corresponds to a reduction in spectrophotometer absorption. This effectuates a descending trend in the calibration curve. The calibration curve derived from the absorption values of the formulated standards is depicted in Fig. 2.

**Fig. 3:**
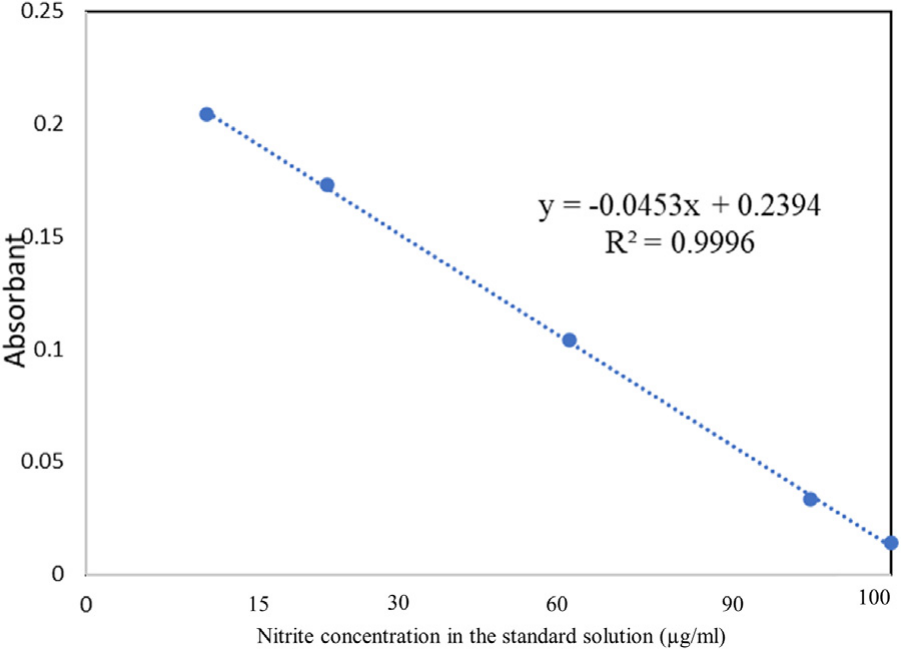
Standard curve of nitrate concentration.

## Validation of the performance of the passive sampler

To validate the data obtained from the passive sampling method, we installed both active and passive samplers at the same location at 2 m height above ground level and at least 15 m distance from any pollution source for 24 h. After 24 h the samplers were collected and transferred to the laboratory, and new samplers, which had been prepared previously, were replaced. After 18 days of repetition, the obtained data were used to compare the performance of the passive sampler. Table 1 shows the mean, standard deviation, minimum, and maximum values of the data obtained from active and passive sampling during 18 days for validation and comparison of the performance of the passive samplers.

**Table 1:** Descriptive statistics of NO_2_ concentration measured by active and passive method data (µg/m^3^).

Method	Average	Standard deviation	Minimum	Maximum
Passive	47.55	7.56	30.33	55.96
Active	47.79	6.81	31.04	53.18

**Fig. 4:**
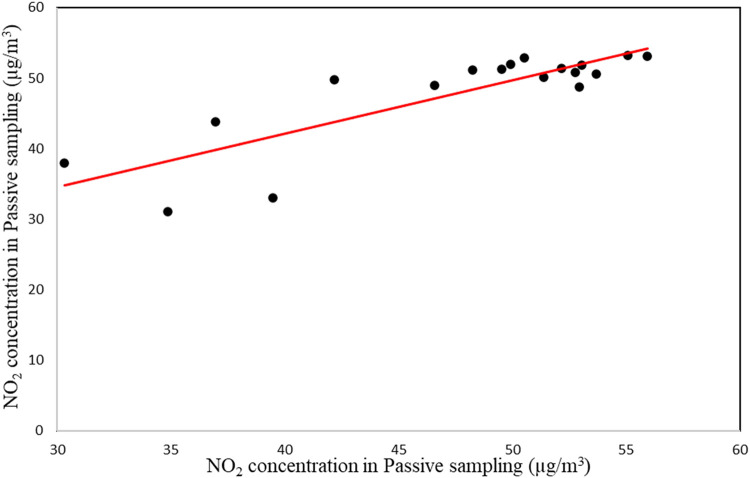
Correlation between active and passive sampling NO2 concentration.

The data measured by developed passive samplers indicated that compared active method indicated the high performance of our developed passive samplers (Fig. 4). The results of the Pearson correlation coefficient indicated that there was a strong correlation between NO_2_ concentrations measured by active and passive methods (r = 0.84, p< 0.01; R^2^ = 70).

## CRediT authorship contribution statement

**Elahe Hasannezhad Estiri:** Resources, Writing – original draft, Writing – review & editing, Methodology. **Abolfazl Rahmani Sani:** Supervision, Writing – review & editing, Methodology. **Afshin Dowlatabadi:** Data curation, Writing – review & editing, Methodology. **Reza Hasannezhad Estiri:** Resources, Writing – review & editing, Methodology. **Mohammad Miri:** Methodology, Supervision, Writing – review & editing.

## Declaration of Competing Interest

The authors declare that they have no known competing financial interests or personal relationships that could have appeared to influence the work reported in this paper.

## Data Availability

Data will be made available on request.
